# Adaptation of influenza A (H7N9) virus in primary human airway epithelial cells

**DOI:** 10.1038/s41598-017-10749-5

**Published:** 2017-09-12

**Authors:** Daniel Tsung-Ning Huang, Chun-Yi Lu, Ya-Hui Chi, Wan-Ling Li, Luan-Yin Chang, Mei-Ju Lai, Jin-Shing Chen, Wen-Ming Hsu, Li-Min Huang

**Affiliations:** 1 Department of Pediatric Infectious Diseases, MacKay Children’s Hospital, Taipei, Taiwan; 2 Department of Medicine, Mackay Medical College, New Taipei City, Taiwan; 3 Department of Pediatrics, National Taiwan University Children’s Hospital, National Taiwan University College of Medicine, Taipei, Taiwan; 4 Institute of Biotechnology and Pharmaceutical Research, National Health Research Institutes, Zhunan, Taiwan; 5 Department of Thoracic Surgery, National Taiwan University Hospital, National Taiwan University College of Medicine, Taipei, Taiwan; 6 Department of Surgery, National Taiwan University Hospital, National Taiwan University College of Medicine, Taipei, Taiwan

## Abstract

Influenza A (H7N9) is an emerging zoonotic pathogen with pandemic potential. To understand its adaptation capability, we examined the genetic changes and cellular responses following serial infections of A (H7N9) in primary human airway epithelial cells (hAECs). After 35 serial passages, six amino acid mutations were found, i.e. HA (R54G, T160A, Q226L, H3 numbering), NA (K289R, or K292R for N2 numbering), NP (V363V/I) and PB2 (L/R332R). The mutations in HA enabled A(H7N9) virus to bind with higher affinity (from 39.2% to 53.4%) to sialic acid α2,6-galactose (SAα2,6-Gal) linked receptors. A greater production of proinflammatory cytokines in hAECs was elicited at later passages together with earlier peaking at 24 hours post infection of IL-6, MIP-1α, and MCP-1 levels. Viral replication capacity in hAECs maintained at similar levels throughout the 35 passages. In conclusion, during the serial infections of hAECs by influenza A(H7N9) virus, enhanced binding of virion to cell receptors with subsequent stronger innate cell response were noted, but no enhancement of viral replication could be observed. This indicates the existence of possible evolutional hurdle for influenza A(H7N9) virus to transmit efficiently from human to human.

## Introduction

In February 2013, avian influenza A(H7N9) virus crossed the species barrier in China and for the first time caused human infections^1^. Since then, the low pathogenicity avian influenza (LPAI) virus has been a persistent public health threat in Southeast Asia, especially in mainland China. The virus is accountable for more than 700 laboratory-confirmed human infections and 300 deaths, with a >30% fatality rate (http://www.fao.org). Epidemiological evidence has linked human cases exposure to live-bird markets, where asymptomatic H7N9 virus-infected chickens appear to be central to this persistent and expanding outbreak^2, 3^. Although human-to-human transmissibility of H7N9 remains restricted, close monitoring for potential pandemic threat via genetic reassortment or mutation is warranted^3, 4^.

Phylogenetic analyses have revealed that the novel H7N9 viruses are likely to emerge from the reassortment of four or more avian influenza A virus strains^1, 5^. Amino acid substitutions in the hemagglutinin (HA) and polymerase basic protein 2 (PB2) viral proteins also enable the virus to overcome the host restriction barrier and infect humans. For example, G186V and Q226L mutations in the HA viral protein could enhance the binding affinity of H7N9 to human-like receptors^6–8^. However, H7N9 viruses possessing leucine or isoleucine at position 226 of HA have been shown to not only bind to α2,6- sialyllactose (SA) but also to α2,3-SA linked receptors, suggesting that the binding of H7N9 virions to human-type receptors might also involve other viral components^8, 9^.

Additional mutations in PB2, such as E627K and D701N, have been shown to increase the replicative ability and virulence in mice, as well as the inter-host transmissibility of avian influenza viruses^10^. By using *ex vivo* human respiratory organ cultures, Chan *et al*. confirmed that PB2-E627K was the most important mutation on PB2 protein for efficient replication of influenza H7N9^11^. These substitutions in PB2 were rapidly selected upon infection of humans with avian H5N1 or H7N9 influenza viruses, adapting the viral polymerase for the mammalian corresponding protein, such as ANP32A^12^.

Similar to A(H5N1) viruses, A(H7N9) influenza viruses have been associated with severe respiratory disease and fatal outcomes in humans, as both viruses are capable of efficient replication in human bronchial and lung tissues^13–15^. Hypercytokinemia has also been reported among severe and fatal cases^16, 17^. Despite the presence of a pressing need to gain a better understanding of the association between severe respiratory disease and A(H7N9) human infection, there are few studies examining the host responses in human airway epithelial cells (hAECs) following the virus infection^13–15^.

Since hAECs are the natural target of influenza virus and also the frontline soldier against respiratory viruses, they are frequently used as tools to evaluate influenza virus localization and virus-induced innate immunity^13–15, 18^. Studying virus adaption and immune responses in hAECs may help to understand copious important issues concerning A(H7N9) virus infection in humans. In this study, we utilized primary hAECs as a model to evaluate viral replication kinetics, amino acid substitutions, and sialic acid binding preference of A(H7N9) virus after sequential passage. We also investigated the elicitation of cytokines and inflammatory mediators following A(H7N9) virus infection in hAECs.

## Results

### Replication of influenza virus A(H7N9) is not altered by serial passage in hAECs

**Figure 1:**
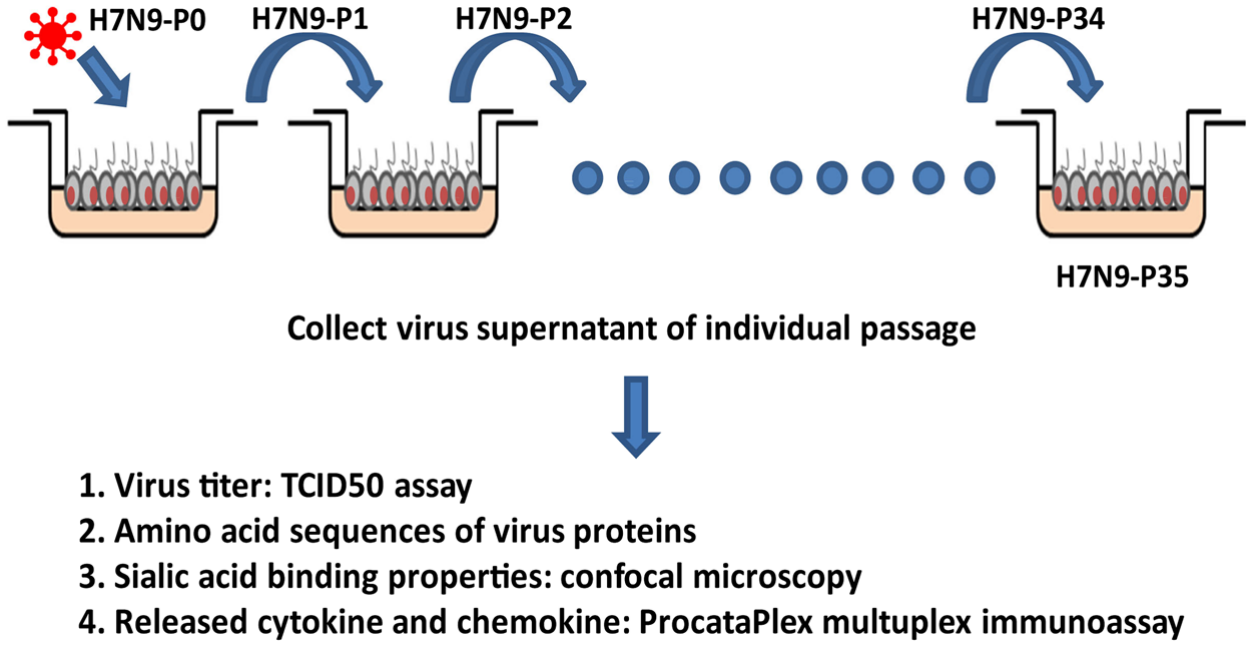
Illustration of how serial passages of A(H7N9) influenza virus were performed on hAECs.

Due to the limited subpassaging ability of the hAECs, three different hAECs series were used in the serial passages of H7N9. Passages 1 (H7N9-P1) to 13 (H7N9-P13) were done on HAE45 (derived from a 53y-old female, non-smoker, right lower lobectomy), whereas passages 14 to 25 and passages 26 to 35 were performed on HAE51 (27y1m, non-smoker, left lower lobectomy), and HAE7 (43y11m, female, non-smoker, right upper lobectomy), respectively (Fig. 1).

**Figure 2:**
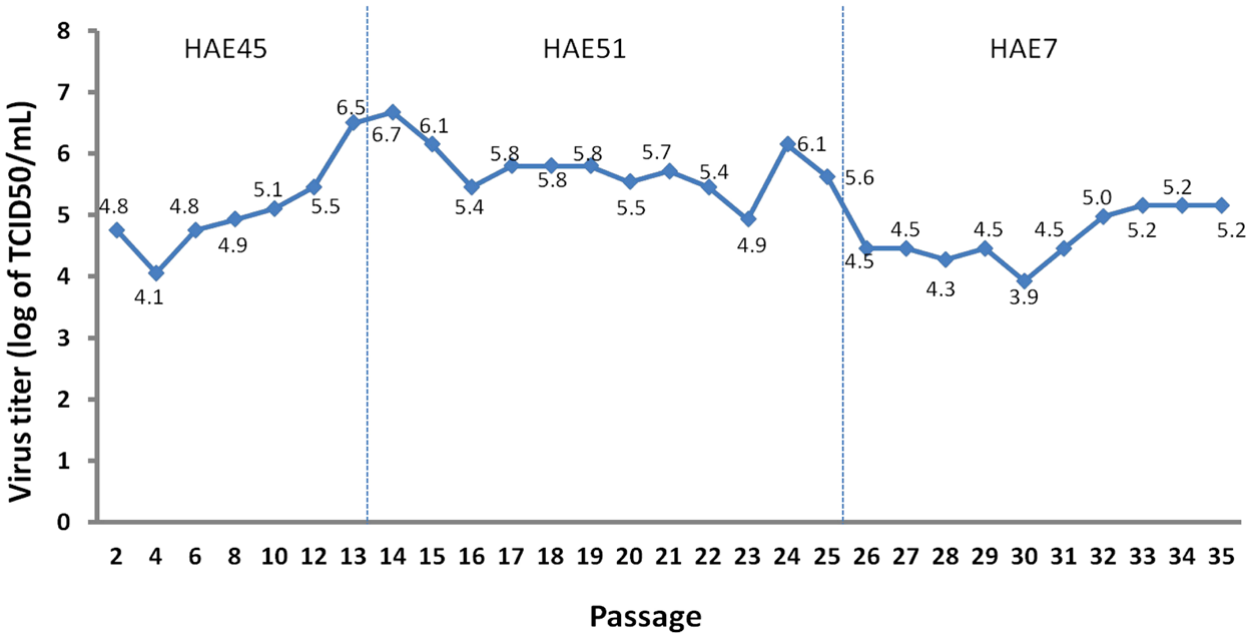
The replication of influenza A(H7N9) virus during serial passage in hAECs. During the 35 passages of A(H7N9) virus in hAECs, viral replication varied but did not change substantially.

We hypothesized that after serial passages in hAECs, influenza A(H7N9) virus would gradually adapt to the environment and undergo mutation, thereby leading to increased viral replication. TCID50 assays showed that the virus titer gradually increased from 4.8 log TCID50/ml to 6.7 log TCID50/ml within the first 14 passages. The titer subsequently dropped to 5.8 log TCID50/ml, remained unaltered for several passages, and then reached a nadir of 3.9 log TCID50/ml at the 30^th^ passage. At the last passage (H7N9-P35), the virus titer was measured at 5.2 log TCID50/ml (Fig. 2). Difference in viral replication pattern was not observed between the three different hAECs series (HAE45, HAE 51, HAE7). These results showed that A(H7N9) virus could replicate well in hAECs, but would not increase its replication via adaptation, if any, in 35 serial passages in 3 different hosts.

### Changes in A(H7N9) virus amino acid sequence after serial passage in hAECs

We examined the alteration of virus amino acid sequences after A(H7N9) virus serial passage in hAECs by subjecting virus supernatants of each passage to RT-PCR and sequence analysis. Influenza virus proteins related to virulence and transmission (HA, NA, PB1, PB1-F2, PB2, PA, PA-X, M1, and NS1) and other viral proteins (NP, M2, NS2) were analyzed. HA and NA genes were sequenced in entirety at all passages. Genes coding for other viral proteins were sequenced at passages 1, 14, 15, and 35.

A total of six changes in the A(H7N9) virus amino acid sequence that occurred during the 35 serial passages were noted (Table 1). While three amino acid mutations occurred in HA (R54G, T160A, Q226L, H3 numbering), others were in NA (K289R, or K292R for N2 numbering), NP (V363V/I), and PB2(L/R332R). Except for NP (V363V/I) and PB2(L/R332R), all other amino acid substitutions were observed in H7N9-P14 and H7N9-P15, corresponding with the timing of the change of the host cells (HAE45 to HAE51), or the first virus titer decrease during serial passage (Table 1, Fig. 2 and Supp Fig). The NP (V363V/I) mutation was found at passage 35, and the PB2(L/R332R) was found in the first passage. A silent mutation occurred in the M1 gene at passage 14 (CAG to CAA at position 75). No sequence change occurred in the coding regions of other viral gene segments.

**Table 1:** Mutations in HA, NA and PB2 amino acid sequences.

HA	NA	NP	PB2	Virulence marker or pathogenic determinant
R54G^c^				Unknown
T160A^c^				Loss of N-glycosylation probably increase virus binding to human type receptor^1^
Q226L^b^				Increase virus binding to human type receptor; enhance the ability of the virus to be transmitted by air^1, 22^
	K289R^c^			Increase susceptibility to Oseltamivir and Zanamivir^10^
		V363V/I^d^		Unknown
			L/R332R^a^	Unknown

Mutations observed in ^a^H7N9-P1, ^b^H7N9-P14, ^c^H7N9-P15, and ^d^H7N9-P35.

### Increased α2,6-sialic acid binding preference for A(H7N9) after serial passage in hAECs

The sialic acid binding specificity of HA is one of the major determinants for controlling viral tropism and host specificity^9^. In general, human influenza viruses have a binding preference for SAα2,6-Gal receptors, whereas avian influenza viruses have a preference for SAα2,3-Gal receptors. We hypothesized that in order to adapt to the environment, A(H7N9) virus would increase its affinity to SAα2,6-Gal receptors, especially after the observed mutations in HA (R54G, T160A, Q226L).

**Figure 3:**
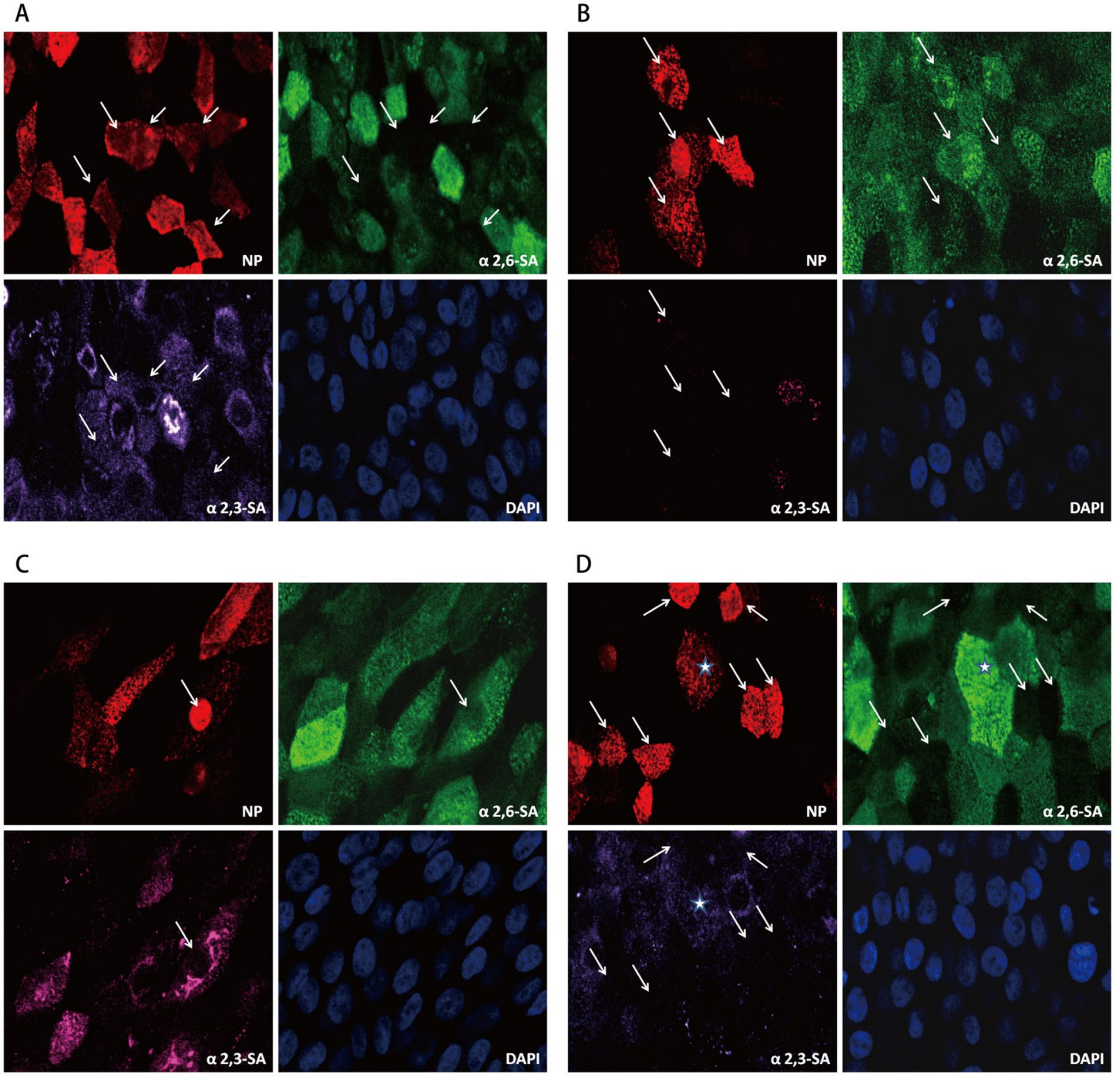
Sialic acid-linked receptors binding properties at individual passage in hAECs. Virus supernatants of passage 1, 15, and 35 were used to infect hAECs. Infected cells were fixed and stained with NP (representing H7N9 virus), Sambucus nigra lectin (SNA, substrate of α2,6-sialic acid), Maackia Amurensis II (MALII, substrate of α2,3-sialic acid) and DAPI at 5 hpi. Each was observed for 8 fields under ZEISS, LSM 510 META Confocal Microscope. Four patterns were observed, including virus located in cells expressing (Fig. 3A) SAα2,3-Gal receptors only, (Fig. 3B) SAα2,6-Gal receptors only, (Fig. 3C) both SAα2,3- and SAα2,6-Gal receptors, and (Fig. 3D) neither SAα2,3- nor SAα2,6-Gal receptors. The white arrows indicate the specific localization panels, and the star signature represents virus infection in cells expressing both SAα2,3- and SAα2,6-Gal receptors.

To verify the influence of HA mutations on sialic acid binding preference, we studied the virus supernatants of H7N9-P1, H7N9-P15, and H7N9-P35 (results shown in Fig. 3). Four infection patterns were observed: A(H7N9) virus infected cells expressing SAα2,3-Gal receptors only (Fig. 3A), SAα2,6-Gal receptors only (Fig. 3B), both SAα2,3- and SAα2,6-Gal receptors (Fig. 3C), and neither SAα2,3- nor SAα2,6-Gal receptors (Fig. 3D). Compared to H7N9-P1, H7N9-P15 maintained similar binding preference to human influenza virus receptors (SAα2,6-Gal receptors) or avian influenza virus receptors (SAα2,3-Gal receptors). However, H7N9-P15 infected a greater proportion of cells expressing neither SAα2,3- nor SAα2,6-Gal receptors, and a smaller proportion of cells expressing both forms of receptors, as compared to H7N9-P1 (Table 2). On the other hand, H7N9-P35 infected more cells expressing human influenza virus receptors (39.2% for H7N9-P1, 37.0% for H7N9-P15, and 53.4% for H7N9-P35) (Table 2). These results implicate that after serial passage in hAECs, A(H7N9) virus developed preference of infecting cells with human influenza virus receptors. However, this binding rate of 53.4% was still lower than the 76.9% (60/78) of seasonal influenza A (H1N1) in a parallel experiment.

**Table 2:** Percentages of sialic acid-linked receptors binding at individual passages (%).

SA^*^ type Passage	SAα2,3-Gal (%)	SAα2,6-Gal (%)	Both (%)	Neither (%)
H7N9-P1	3.1(3/97)	39.2(38/97)	15.5(15/97)	42.3(41/97)
H7N9-P15	1.2(1/81)	37.0(30/81)	4.9(4/81)	56.8(46/81)
H7N9-P35	3.8(5/133)	53.4(71/133)	10.5(14/133)	32.3(43/133)

^*^SA, sialic acid.

### Stronger immune responses induced by A(H7N9) virus after serial passage in hAECs

**Figure 4:**
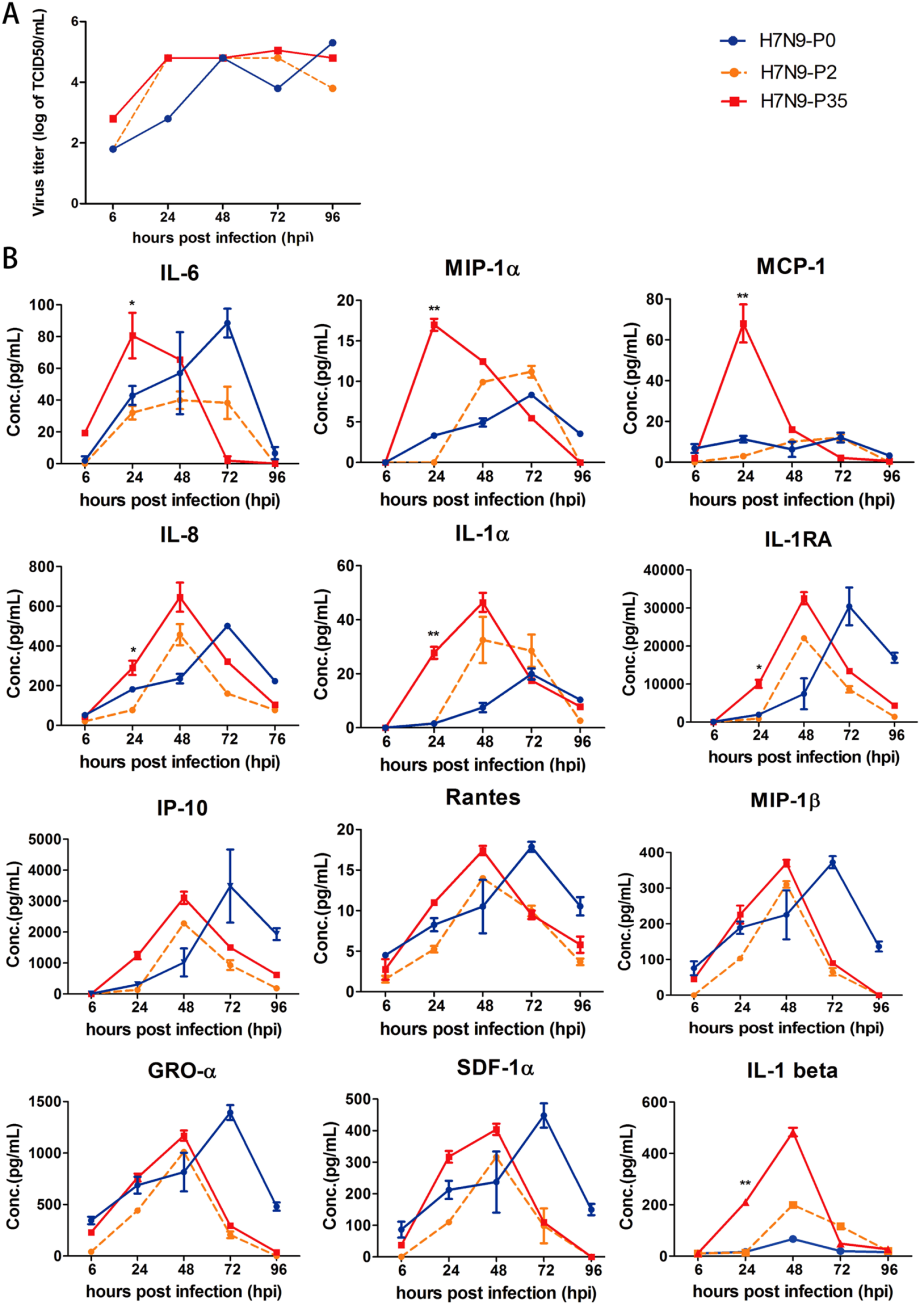
Cytokine responses to H7N9-P0, H7N9-P2 and H7N9-P35 infection in hAECs. Virus supernatants were collected at 6, 24, 48, 72 and 96 hpi and analyzed by TCID50 assay for virus titer determination and procataplex immunoassay for cytokines and chemokines. (Figure 4A) Virus replication kinetics of H7N9-P0, H7N9-P2 and H7N9-P35 were similar. (Figure 4B) Cytokine and chemokine expression panels at 0(H7N9-P0), 2nd (H7N9-P2) and 35th (H7N9-P35) passages in hAECs. Blue line: H7N9-P0, Orange line: H7N9-P2, Red line: H7N9-P35. Statistics method: Two-way ANOVA. *P < 0.01, **P < 0.001 at 24 hpi.

To study the local immune responses after serial passages, we infected HAE9 derived from left upper lobectomy of a 54y1m male nonsmoker with 100 TCID50 of H7N9-P0, H7N9-P2 and H7N9-P35. Results showed similar replicating patterns among the three viruses at 6, 24, 48, 72 and 96 hpi (Fig. 4A). Thirty-four different cytokines and chemokines were analyzed by ProcartaPlex multiplex immunoassay at 6, 24, 48, 72 and 96 hpi. Levels of twelve cytokines and chemokines in the apical supernatant of hAECs were elevated following A(H7N9) virus infection, including GRO-α, MIP1-α, MIP1-β, SDF-1α, RANTES, IL-1α, IP10, IL-1RA, MCP1, IL-8, IL-6, and IL-1β. With the exception of IL-1RA, these cytokines and chemokines mediate proinflammatory effects via different pathways. These proinflammatory cytokines are induced by TLR3 and RIG-1 in epithelial cells, which are triggered by viral RNAs, causing subsequent tissue inflammation. In addition, IL-8, MCP1, RANTES, and IP10 are responsible for chemoattraction of monocytes and macrophages^19^.

Compared to H7N9-P0, H7N9-P35 consistently induced stronger local immune responses than H7N9-P2 by eliciting higher cytokine levels, especially IL-6, MIP-1α, MCP-1, IL-8, IL-1α, IL-1RA, and IL-1β at 24 hpi (Fig. 4B). Notably, IL-6, MIP-1α, and MCP-1 levels peaked at 24 hpi. The results demonstrate that after serial passage in hAECs, A(H7N9) virus is capable of inducing faster and stronger local immune responses.

## Discussion

Adaptation is believed to be a driving force in evolution, whereby organisms, including viruses, are selected in nature because of increased fitness conferred by gene mutations^20^. Although multiple studies have examined the infectivity of A(H7N9) virus infection in human bronchial epithelial cells, we are the first to examine the adaptation of this virus in hAECs through serial passage^13–15^. In this study, we found that A(H7N9) virus effectively infected primary hAECs and maintained stable replication after 35 serial passages. Six amino acid mutations were found on HA, NA, NP, and PB2 after serial passage in hAECs; however, these changes did not significantly enhance viral infectivity. On the other hand, the mutations in HA enabled A(H7N9) virus to bind with higher affinity to human influenza virus receptors. After serial passage, A(H7N9) virus infection elicited a greater production of proinflammatory cytokines in hAEC, compared with that of the first generation virus-infected cells.

Among the 6 amino acid mutations, some have documented significance in virulence or pathogenic determinant, while others do not (Table 1). Positions 54, 160, and 226 (H3 numbering) of the HA protein are all located in the receptor binding domain^21^. Q226L substitution of the HA protein has been extensively studied, and found to substantially enhance influenza virus binding capacity to human type receptors^1, 6, 22^. T160A causes the loss of a glycosylation site on the 150-loop, which might decrease the affinity of A(H7N9) virus to α-2,3 avian-like receptors^1^. The significance of R54G mutation remains uncertain.

Contrary to the famous mammalian cell-adapting PB2-E627K and D107N mutations in the NLS domain^11^, the L332R mutation in PB2 we found in this study was located in the Cap-binding domain^23^. In studying mouse adaptation of an H5N1 virus isolated from duck, Li *et al*. identified an entire series of mutations (L89V, G309D, T339K, R477G, I495V and A676T)^24^, some of which were located in the Cap-binding domain. However, the significance of substitution PB2-L332R in our study remains to be elucidated.

Mutation H274K (N2 numbering) within the neuraminidase (NA) gene has been previously reported to confer a very high level of resistance to oseltamivir without compromising viral fitness in influenza viruses^25^. Another gene mutation, R289K (R292K, N2 numbering), found in the first reported case of H7N9 (/Shanghai/1/2013) in China, has also been shown to reduce oseltamivir and zanamivir susceptibility^10^. Conformational changes induced by the mutation R289K cause the loss of a number of hydrogen bonds between the inhibitors and the A(H7N9) viral neuraminidase complexes^26^. Of note, the 289K reverted back to Arg after 14 drug-free serial passage of A(H7N9) virus in hAECs, suggesting that the NA protein is functionally more comfortable with 289 R without drug challenge. For H7 influenza viruses, two mutations (i.e. HA Q226L and T160A) found in almost all Chinese H7N9 isolates so far have been described to enhance SAα2,6-Gal receptors binding^1, 5, 9^. This phenomenon is further confirmed by the immunofluorescent staining and confocal microscopy scanning results in our study, as the percentage of virus binding to SAα2,6-Gal receptors significantly increased from 39.2% (H7N9-P1) to 53.4% in the later passage (H7N9-P35) after the appearance of Q226L and T160A mutations in H7N9-P14 and H7N9-P15 (Table 2). We observed that H7N9-P35 with an enhanced binding to human-type receptor did not replicate much better than that of H7N9-P2 and H7N9-P15 with less affinity to SAα2,6. It suggests that there is another known rate-limiting step to restrict viral replication. The fact that we did not detect mutations in other viral replication genes other than PB2(L/R332R) was in line with this explanation.

The lack of changes in the binding preference to SAα2,3-Gal receptors suggests that adaptive mutations had no effect on the binding affinity to avian type receptors. This result is in line with previous studies using the glycan microarray method to determine the sialic acid binding preference for H7N9 virus^27–29^. As to SAα2,6-Gal receptors, Xu *et al*. found that even bearing Q226L mutation such as A/Shanghai/02/2013(H7N9), the H7 HA is predominantly specific for avian-type (α2-3) receptors with only weak binding to human receptors^28^. The author suggested that the intrinsic weak avidity of H7 to human receptors in glycan receptor assays is sometimes exaggerated, especially in studies with whole virus. The exaggeration could be explained by the preferential cleavage of the avian-type receptors by the neuraminidase and the high valency of HA on the virus that can amplify binding to human receptors of weaker affinity^30, 31^. Instead of using glycan microarray, we employed the lectin histochemistry on cultured hAECs to determine the α2,3- and α2,6- sialic acid binding preference in our study. By this mean we could mimic the real dynamic of H7N9 virus approaching the receptors on human respiratory cells, and also incorporate the influence of the viral neuraminidase. Interestingly, our result showed that the HA Q226L did contribute to a better binding to human SAα2,6-Gal receptors of H7N9 virus, because the increment of binding ratio is concomitant with the timing of mutation, which were observed in H7N9-P14 and H7N9-P15 (Table 2 and Supp Fig). Since cultured hAECs express little α2,3-sialic acid, which might be further cleaved by N9 neuraminidase, it is not surprising that the ratio of virus binding to cells expressing purely SAα2,3-Gal receptors was very low in our study (Table 2).

The reason why a large percentage of hAECs without α2,6- or α2,3-sialic acid staining was infected by A(H7N9) virus may be in part due to the unsatisfactory staining technique of hAECs, and also the influence of N9 neuraminidase cleavage. Those unstained cells might express insufficient numbers of SAα2,6- and SAα2,3-Gal receptors too low to be detected by immunofluorescence staining. Under experimental setting similar to ours, influenza viruses were found to successfully infect host cells, in which all sialic acid residues have been cleaved by a broad-spectrum neuraminidase. This observation thereby led the authors to conclude that the presence of even a minimal amount of sialic acid could have allowed influenza viruses to gain cellular entry^18^.

As we know, the determinants of efficient airborne transmission of influenza A viruses between mammals are multiple, including the adaptation of HA binding preference, the stabilization of fusion between the endosomal and viral membranes, and the enhancement of transcription of vRNA, mRNA and cRNA^32^. In many respiratory droplet transmission studies, higher viral titers collected from animal airways do not promise a better viral transmissibility in mammals^33, 34^. However, the correlation between the viral titers and transmission still exist to a certain extent in other studies^35^. In our data, regardless of the six adaptive amino acid mutations occurred, the influenza A(H7N9) virus did not show a significant enhancement of virus infectivity. This indicates the existence of a possible evolutional hurdle for influenza A(H7N9) virus to transmit efficiently from human to human.

In our study, levels of 12 cytokines and chemokines in the apical supernatant of hAECs were elevated following A(H7N9) virus infection, including GRO-α, MIP1-α, MIP1-β, SDF-1α, RANTES, IL-1α, IP10, IL-1RA, MCP1, IL-8, IL-6, and IL-1β. These findings not only support previous findings^13–17^, but further provide a detailed description of hAEC-specific cytokine profile for future studies. We also found that after serial passages, the adapted H7N9-P35 could induce faster and greater local immune responses than H7N9-P2 in hAECs, especially for IL-6, MIP-1α, and MCP-1, whose levels peaked at 24 hpi (Fig. 4B). A plausible explanation may be that the improved adaptation of H7N9-P35 to hAECs enhanced viral entry and increased induction of proinflammatory cytokines. Apical secretion of these cytokines and chemokines by hAECs may lead to the recruitment of inflammatory cells into airways lumen that could subsequently cause narrowing of air spaces, increasing in ventilation-perfusion mismatch, and deterioration of lung function^1, 16^. The perturbation of chemokine and cytokine responses has also been considered to be associated with severe human H5N1 and H7N9 infections^1, 16, 21^.

Innate immunity, shown as inflammatory cytokine responses, played a pathogenic role in H5N1 and H7N9 infections^15–17, 36, 37^. Higher levels of inflammatory cytokines were correlated with adverse clinical outcome. However, it is also important to note that in H7N9 patients, neither the clinical outcome nor all the cytokine levels correlate with the viral load in patients’ throats^36^. This indicates that although a good viral entry is the first key for H7N9 virus to infect human, a better replication does not fully explain the severity of the disease. According to our data, we propose that the maybe the improved adaptation of H7N9 virus to hAECs facilitates greater viral entry, leading to a higher level of induced proinflammatory cytokines which are responsible for the poor clinical outcome^37, 38^. However, the virus still cannot replicate efficiently.

Influenza A viruses have been previously reported to induce IL-6 *in vitro* in the transformed bronchial epithelial cell line (NCI-H292)^39^. IL-6 is a multifunctional cytokine that can regulate immune and inflammatory responses involved in the activation, growth and differentiation of T-cells, and can contribute to T-cell-mediated inflammatory reactions^40^. Physiological analysis of sickness behavior of mice following influenza infection revealed that virus-associated body temperature and motor activity decreases were significantly attenuated in mice lacking IL-6, compared to wild-type infected mice^41^. Furthermore, in experimental human infection, IL-6 levels in both serum and nasopharyngeal lavage were found to correlate with symptom scores and temperature values^42^. MIP-1α, also called chemokine (C-C motif) ligand 3, exhibits chemotactic properties for eosinophils, monocytes, and lymphocytes^42^. Studies using homozygous MIP-1α mutant (−/−) mice showed reduced pneumonitis and delayed clearance of virus compared to infected +/+ mice^43^. MCP1 is a chemokine that attracts macrophages and mediates inflammatory response via recruitment of circulating leukocytes to the inflamed tissue^44^. Fatal outcomes following human infection with avian influenza A virus (H5N1) have been associated with MCP1 elevation in the peripheral blood^44^. Compared to H7N9-P2, H7N9-P35 provoked earlier elevation of IL-6, MIP-1α, and MCP1. However, the mechanism contributing to such a difference is still unknown and requires further research. One limitation of this study was the subpassaging ability of hAECs which required us to use samples from different hosts to complete the serial passage. The benefit of such maneuver was that these experiments reflected the real-life scenario of A(H7N9) virus passing through three different human hosts. In this study, the amino acid substitutions in HA, NA, and M1 silent were observed at H7N9-P14 and H7N9-P15, coinciding with the timing of change of the host cells (from HAE45 to HAE51). This finding is of future research interest since it suggests that some infected hosts may provide a greater stress on the evolution of influenza virus than others by inducing difference immune responses.

## Conclusions

Six amino acid mutations were found in A(H7N9) virus during the process of adapting to human cells, including three in HA (R54G, T160A, Q226L, H3 numbering), one in NA (K289R, or K292R for N2 numbering), one in NP (V363V/I), and one in PB2(L/R332R). Our findings showcase the extent of influenza A (H7N9) virus adaptation in human cells and can provide information for the design of vaccine and management of potential outbreak. The adapted virus can bind better to human receptor and has the potential to cause more severe diseases. However, it still cannot replicate efficiently.

## Materials and Methods

### Viruses

The A (H7N9) influenza virus was isolated from the first imported H7N9 human case in Taiwan (A/Taiwan/1/2013(H7N9))^45^. Virus was amplified in MDCK (Madin-Darby canine kidney) cells, and virus titer was determined by TCID50 assay.

### Primary human airway epithelial cell (hAEC) culture

Normal primary hAECs were isolated from lung cancer patients who underwent lobectomy surgery at the Department of Surgery, National Taiwan University Hospital (NTUH), Taipei, Taiwan. Informed consent was obtained from all patients. Isolated tissues were digested with protease XIV-DNase I (Sigma) and the following additives (Sigma): penicillin G sulfate (100 units/ml), streptomycin sulfate (100 μg/ml), amphotericin B (1.25 μg/ml), gentamicin (50 μg/ml), and nystatin (100 units/ml), and immersed in minimal essential medium (MEM; Invitrogen) at 4 °C for 24 to 48 hours. After cell dissociation, the hAECs were maintained for one or two serial passages as a monolayer in bronchial epithelial cell serum-free growth medium (BEGM), which is LHC basal medium (Invitrogen) supplemented with the required additives (Sigma Aldrich). BEGM was refreshed at 2- or 3-day intervals. Upon reaching 80% confluence, hAECs were passaged to form pseudostratified hAEC cultures as described elsewhere^46^. Cultures were maintained at air-liquid interface (ALI) for 4 to 6 weeks for cellular differentiation. Prior to the experiments, all cultures were maintained at 37 °C in a 5% CO_2_ incubator.

### Backgrounds of hAECs used in each experiment

Due to the the limited subpassaging ability of the hAECs, three different hAECs series were used in the serial passages of H7N9: HAE45 (derived from a 53y-old female, non-smoker, right lower lobectomy), HAE51 (27y1m, non-smoker, left lower lobectomy), and HAE7 (43y11m, female, non-smoker, right upper lobectomy). Time course analysis of cytokine expression at 3 different passages (H7N9-P0, H7N9-P2 and H7N9-P35) was performed on HAE9 (54y1m, male, non smoker, left upper lobectomy).

### H7N9 virus serial infection and virus supernatant collection

100 TCID50 of the amplified virus (H7N9-P0) was diluted in 200 μl HBSS and was directly used to inoculate the apical surface of pseudostratified hAECs. After incubation at 37 °C in a 5% CO_2_ incubator for 1 hour, the inoculated virus supernatant was isolated and washed with HBSS to remove the attached viruses. Inoculated cultures were maintained at 37 °C in a 5% CO_2_ incubator. After 48 hours post infection (hpi), apical virus supernatants were harvested by adding 300 μl HBSS to the apical surface and incubating for 30 min at 37 °C under 5% CO_2_. Ten μl of each of the collected virus supernatant were used to infect the next generation hAECs, and serial passage was repeated 35 times. Passages 1 (H7N9-P1) to 13 (H7N9-P13) were done on HAE45, whereas passages 14 to 25 and passages 26 to 35 were performed on HAE51, and HAE7, respectively (Fig. 1). Virus supernatants (200 μl) was transferred to 300 μl lysis buffer for total nucleic acid extraction, and the remaining supernatants were used for virus titer determination.

### Virus titer determination

Virus titer from each A (H7N9) passage was determined by the TCID50 assay. To prepare for the assay, 3 × 10^4^ MDCK cells/well were seeded in the 96-well plate. After 24 hours, the virus supernatants collected from hHACs were serially diluted at 1:10. MDCK cells were infected by the diluted supernatants at aliquots of 50 μl and incubated at 37 °C 5% CO_2_ for 1 hour. The infected cells were washed with PBS and cultured in MEM with 2% FBS and 1 μg/ml TPCK-trypsin. Cells were later incubated in a 37 °C, 5% CO_2_ incubator for 24 hours and then fixed in 80% acetone for 10 min. Fixed cells were washed twice with wash buffer and incubated with anti NP monoclonal antibody (1:1500, Santacruz) at 37 °C for 1 hour. Cells were washed with wash buffer 3 times and incubated with anti-mouse IgG-HRP (1:2000, Jackson) at 37 °C for 1 hour. Cells were washed with wash buffer 6 times, reacted with OPD substrate, and stopped by 1N of sulfuric acid. OD values at 492nm were read by EZRead 400 microplate (biochrom). TCID50 values were calculated as per standard protocol.

### Virus amino acid sequence analysis

At each passage, the total nucleic acid of the H7N9 virus supernatant was extracted from the apical compartment by MagNA pure LC total nucleic acid isolation kit (Roche). Total nucleic acids were reverse transcriptased by RTase (Qiagen). cDNAs underwent PCR by ExTag (Qiagen). PCR program are described as follow: 94 °C-1 min, 35 cycles of 94 °C-40 s, 45–50 °C-40 s, 72 °C-2min, and finally 72 °C, 8 min and 4 °C. The forward and reverse primers for each virus CDS are described below, **HA:** 5′-ATG AAC ACT CAA ATC CTG GTA-3′ and 5′-TAT ACA AAT AGT GCA CCG CA-3′. **NA:** 5′-ATG AAT CCA AAT CAG AAG ATT C-3′ and 5′-GAG GAA GTA CTC TAT TTT AGC-3′. **PB-1:** 5′-ATG GAT GTC AAT CCG ACT TTA C-3′ and 5′-CTA TTT TTG CCG TCT GAG CTC TTC-3′. **PB2:** 5′-ATG GAA AGA ATA AAA GAA CTA AGA GAT TTG A-3′ and 5′-TTA ATT GAT GGC CAT CCG AAT C-3′. **PA:** 5′-ATG GAA GAC TTT GTG CGA CA-3′ and 5′-CTA GCT TAG TGC ATG TGT GA-3′. **NP:** 5′-ATG GCG TCT CAA GGC ACC AAA-3′ and 5′-TCA ATT GTC ATA CTC CTC TGC A-3′. **M1/M2:** 5′-ATG AGT CTT TTA ACC GAG GTC GAA-3′ and 5′-TCA CTT GAA CCG CTG CAG TTG CA-3′. **PA-X:** 5′-ATG GAA GAC TTT GTG CGA CAG T-3′ and 5′-TCA CTT CTT TTG ACA TCT GAG AAA. **PB1-F2:** 5′-ATG GAA CAG GAA CAG GAT ACA and 5′-TCA GTT TAT CCA CTC TTG TTT GC-3′. **NS1/NS2:** 5′-ATG GAT TCC AAT ACT GTG TCA A-3′ and 5′-CTA CTT TGT AGA GAG TGG AGA TC-3′.

### Sialic acid staining

Virus supernatants from serial passage 1 (H7N9-P1), 15 (H7N9-P15) and 35 (H7N9-P35) in hAECs were used for sialic acid staining study. Infected hAECs were fixed at 5 hpi with 4% paraformaldehyde at room temperature for 20 minutes. Fixed cells were washed with PBS, permeabilized with 0.3% tritonX-100 for 5 minutes and blocked with 5% BSA-PBS at room temperature for 1 hour. Cells were stained with mouse anti-influenza A NP monoclonal antibody (1:500, Santacruz), FITC conjugated *Sambucus nigra* lectin (SNA, substrate of α2,6-sialic acid. 1:100, Vector Lab), and Biotin labeled *Maackia Amurensis* lectin II (MALII, substrate of α2,3-sialic acid. 1:50, Vector Lab) at 37 °C for 1.5 hours. Cells were washed with PBS and stained with fluorescent labeled secondary antibodies, including Alex568 conjugated goat anti-mouse IgG (1:1000, Invitrogrn) and Cy5 conjugated Streptavidin (1:1000, Invitrogrn) at 37 °C for 1 hour. Cells were washed with PBS and covered with mounting medium with DAPI (Biotium). Stained cells were preserved on cover slide and each slide was observed for 8 fields under ZEISS, LSM 510 META confocal microscope.

### Cytokine and inflammatory mediator analysis

hAECs were infected with 100 TCID50 of H7N9-P0, H7N9-P2, H7N9-P35, and human A(H1N1). Following virus infection at 6, 24, 48, 72 and 96 hpi, virus supernatants from the apical compartment of the hAECs were collected. Virus titers were determined by TCID50 assay as previously described. Virus supernatants were inactivated by 0.1% paraformaldehyde at room temperature for 3 hours and subject to immune response determination. Cytokines and chemokines concentrations were analyzed by ProcartaPlex Multiplex Immunoassay (Affymetrix, eBioscience). Time course cytokine analysis (H7N9-P2 and H7N9-P35) was done on HAE9 (54y1m, male, nonsmoker, left upper lobectomy). A total of 34 cytokines and chemokines were analyzed, including IFN-γ, IFN-α, TNF-α, TNF-β, GM-CSF, Eotaxin, GRO- α, IP-10, RANTES, MIP-1α, MIP-1β, SDF-1α, MCP-1, IL-1α, IL-1β, IL-2, IL-1RA, IL-4, IL-5, IL-6, IL-7, IL-8, IL-9, IL-10, IL-12p70, IL-13, IL-15, IL-17A, IL-18, IL-21, IL-22, IL-23, IL-27, IL-31.

All methods involving live H7N9 virus were performed in a biosafety level 3 lab and accordance with the P3 lab guidelines and regulations of National Taiwan University Medical College. All methods were performed in accordance with the relevant guidelines and regulations.

### Ethical approval

The study was approved by the National Taiwan University Hospital (NTUH) Research Ethics Committee (number: 201309062RINB).

## Electronic supplementary material

Additional Information
