# Supplementary material for: Adaptation of influenza A (H7N9) virus in primary human airway epithelial cells
Source: Sci Rep. 2017 Sep 12;7:11300. doi: 10.1038/s41598-017-10749-5 (PMC5595892; doi:10.1038/s41598-017-10749-5)
Supplement: Supplementary file 1 — Additional Information [file 41598_2017_10749_MOESM1_ESM.pdf]

Adaptation of influenza A (H7N9) virus in primary human airway epithelial cells

Daniel Tsung-Ning Huang[1,2 ¶], Chun-Yi Lu[3¶], Ya-Hui Chi[4], Wan-Ling Li[3], Luan-Yin Chang[3], Mei-Ju Lai[3], Jin-Shing Chen[5], Men-Ming Hsu[6], Li-Min Huang[3*]

[1]Department of Pediatric Infectious Diseases, MacKay Children's Hospital, Taipei, Taiwan

[2] Department of Medicine, Mackay Medical College, New Taipei City, Taiwan

[3]Department of Pediatrics, National Taiwan University Children's Hospital, National Taiwan University College of Medicine, Taipei, Taiwan

[4]Institute of Biotechnology and Pharmaceutical Research, National Health Research Institutes, Zhunan, Taiwan

[5]Department of Thoracic Surgery, National Taiwan University Hospital, National Taiwan University College of Medicine, Taipei, Taiwan

[6]Department of Surgery, National Taiwan University Hospital, National Taiwan University College of Medicine, Taipei, Taiwan

*Corresponding author

E-mail: lmhuang@ntu.edu.tw (LMH)

[¶]These authors contributed equally to this work.

.

Additional Information

Supplementary information accompanies this paper at http://www.nature.com/srep

Competing financial interests: The authors declare no competing financial interests.

Supporting information

Supp Fig. Amino acid point mutations in HA, NA, NP, and PB2 after serial passage of influenza A(H7N9) virus in hAECs.

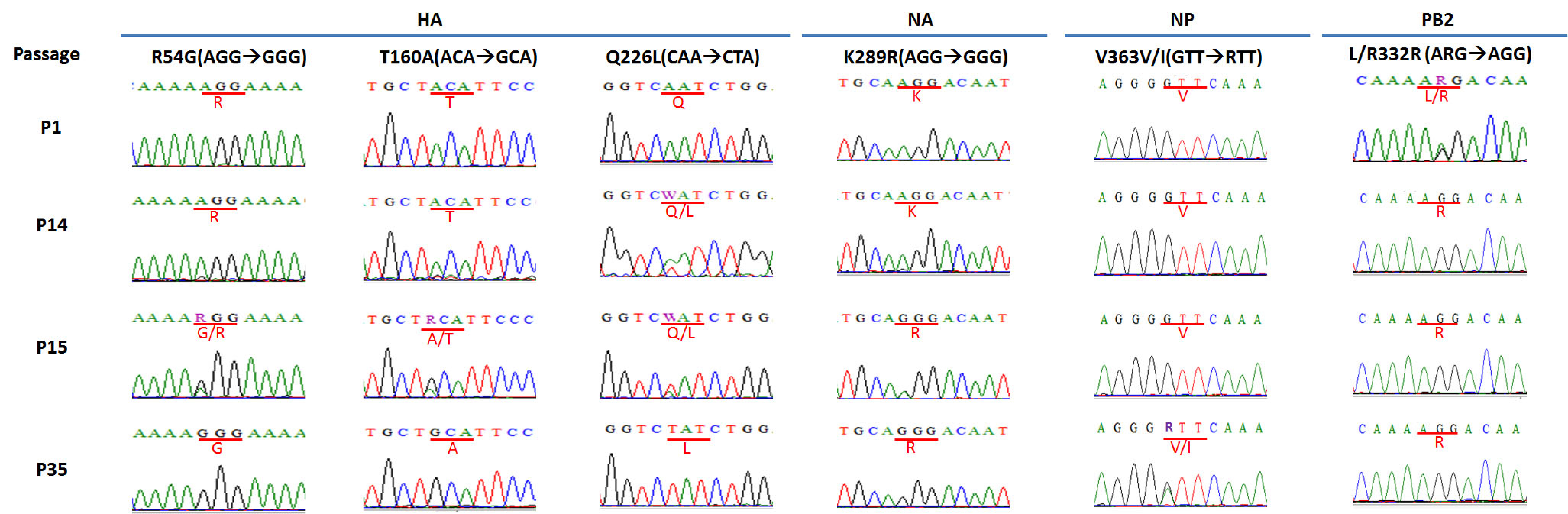

HA
NA
NP
PB2
Passage
R54G(AGG→GGG)
T160A(ACA→GCA)
Q226L(CAA→CTA)
K289R(AGG→GGG)
V363V/I(GTT→RTT)
L/R332R (ARG→AGG)
P1
P14
P15
P35
